# Personality and Situation Predictors of Consistent Eating Patterns

**DOI:** 10.1371/journal.pone.0144134

**Published:** 2015-12-03

**Authors:** Uku Vainik, Laurette Dubé, Ji Lu, Lesley K. Fellows

**Affiliations:** 1 Montreal Neurological Institute, Department of Neurology & Neurosurgery, McGill University, Montréal, Québec, Canada; 2 Institute of Psychology, University of Tartu, Tartu, Estonia; 3 Desautels Faculty of Management, McGill University, Montréal, Québec, Canada; 4 Faculty of Agriculture, Dalhousie University, Truro, Nova Scotia, Canada; Duke University, UNITED STATES

## Abstract

**Introduction:**

A consistent eating style might be beneficial to avoid overeating in a food-rich environment. Eating consistency entails maintaining a similar dietary pattern across different eating situations. This construct is relatively under-studied, but the available evidence suggests that eating consistency supports successful weight maintenance and decreases risk for metabolic syndrome and cardiovascular disease. Yet, personality and situation predictors of consistency have not been studied.

**Methods:**

A community-based sample of 164 women completed various personality tests, and 139 of them also reported their eating behaviour 6 times/day over 10 observational days. We focused on observations with meals (breakfast, lunch, or dinner). The participants indicated if their momentary eating patterns were consistent with their own baseline eating patterns in terms of healthiness or size of the meal. Further, participants described various characteristics of each eating situation.

**Results:**

Eating consistency was positively predicted by trait self-control. Eating consistency was undermined by eating in the evening, eating with others, eating away from home, having consumed alcohol and having undertaken physical exercise. Interactions emerged between personality traits and situations, including punishment sensitivity, restraint, physical activity and alcohol consumption.

**Conclusion:**

Trait self-control and several eating situation variables were related to eating consistency. These findings provide a starting point for targeting interventions to improve consistency, suggesting that a focus on self-control skills, together with addressing contextual factors such as social situations and time of day, may be most promising. This work is a first step to provide people with the tools they need to maintain a consistently healthy lifestyle in a food-rich environment.

## Introduction

Consistent behaviour can be life-saving. For example, when driving a car, the goal is to stay consistently in the lane. That task can be quite challenging—single vehicle road departure accidents cause a third of all driving fatalities in Europe and the US [1,2]. Factors undermining driving consistency depend on both the driver and the situation; these factors include driver inattentiveness and fatigue, driving under the influence of alcohol, weather conditions, and having several people in car [3]. Modern cars monitor these factors with electronic assistance systems to help drivers stay on track (e.g., [4,5–9]).

Consistent behaviour can be just as beneficial in eating regulation. One example of consistency is having meals at similar times of a day. This has been recommended as a part of the 10 Top Tips intervention for weight loss, as consistency is expected to encourage healthy habit development [10]. In addition, eating behaviors can be consistent in terms of maintaining a similar dietary pattern across different eating episodes. This does not imply having exactly the same food every day. Rather, people can have varied individual food items as recommended, for example, in the American Dietary Guidelines [11], but their day-to-day meals are consistent in terms of energy content and healthiness. Consistency in these dimensions is likely to support healthy eating [12–14], and are the focus of the current study.

Being consistent with regards to food intake might be particularly difficult in the current food environment, in which cheap calories are widely available [15–18], and aggressively marketed [19]. Humans implicitly value higher-calorie foods, so the availability of food and its marketing might divert people from their typical eating patterns. While thriftily acting upon food cues, and thus deviating from typical eating patterns, might have been beneficial in a distant past marked by food scarcity ([20,21], c.f., [22]), in today’s world full of appetitive temptations, over-reliance on external food cues can be a disadvantage [23–26].

There is some evidence that the ability to maintain consistent eating regardless of the food environment may be adaptive. For example, Pachucki [27] recently compared the obesity risk of participants with different diet trajectories. Body mass index (BMI) levels were largely the same for participants with consistent trajectories or participants making a healthy change in diet. Participants with inconsistent trajectories or making an unhealthy change in diet were considerably more likely to have a higher BMI (Table 5 in [27]). Another study concluded that participants eating a similar number of calories each day have lower body fat percentage, lower fat and energy intake compared to those with more inconsistent caloric intake [28]. Self-reported eating consistency is associated with an array of more general health benefits, including successful weight maintenance [29–31] and lower risk for metabolic syndrome [32].

Despite the health benefits of eating consistency, the determinants of this behaviour are largely unknown. Understanding any behaviour requires considering both person-related and environment-related variables [33]. This approach has already proved fruitful to explain driving consistency [3]. Therefore, the goal of the current study is determine the person and situation factors predicting eating consistency. Knowing the determinants of eating inconsistency can lead to more informed methods for designing interventions.

Person factors include any characteristic of the person, including age, body weight, and psychological traits. One study on eating consistency suggests that males and older people are more consistent [34]. However, more complex psychological person factors of eating consistency have not been studied. Usually, these person factors are analysed in the framework of personality traits—aggregate summaries of what people want, say, do, feel, or believe [35,36]. Based on personality-consistency studies from other behavioural domains [37–40] and our own recent review of personality predictors of BMI [41] we hypothesize that traits facilitating reactivity to environment (punishment sensitivity, reward sensitivity) will be associated with decreased consistency, and traits associated with self-control will be associated with increased consistency.

Situations refer to features of the environment that influence human behaviour. Many studies have demonstrated that changes in situations can result in surprisingly robust changes in eating behaviour (e.g., [42,43,44]). However, the evidence is quite scattered—unlike in the field of personality, a coherent, widely-accepted taxonomy of eating situations has yet to emerge (c.f., [45,46]). In the current study, we focus on a few situation factors that have been shown to influence eating: eating outside the home, eating with others and alcohol consumption have all been shown to increase food consumption (e.g., [47,48–50]). These situations involve being in novel situations, and having fewer attentional resources available for conscious control (e.g., [51]). As a result, one could expect that in these situations a more automatic reaction to environmental food signals could trigger a reduction in eating consistency. Physical activity has also been suggested to increase palatability of food and change food intake, especially in women [52,53]. However, such changes in intake might not be necessarily maladaptive. According to a recent interpretation, being physically active leads to better responsiveness to one’s energy needs [54]. Therefore, physical activity could cause eating inconsistency, but this might be an appropriate response. The same logic applies for physical work (e.g., [55]).

Other studies suggest an influence of time. There is some evidence showing that people are less consistent on weekends than on weekdays (see [56] for an overview). Consistency could also be lower in the evening, as Baumeister and Heatherton propose that people are more likely to break their diet later during the day because they are fatigued and have fewer self-control resources available [57]. This view has been challenged by Bandura who proposes that self-regulation failure is instead caused by the particular situations the person is in (e.g., drinking alcohol). The evening effect emerges because such situations are more common later in the day [58].

Finally, some of the aforementioned situations are also known to interact with personality traits. For instance, restrained persons are known to consume less food when they have consumed alcohol [49,59]. Similarly, overweight and restrained persons tend not to increase their food intake after exercise [53]. Therefore, restraint could reduce the effects of alcohol consumption and physical activity on consistency.

In sum, we believe that eating consistency is an under-studied behavioural phenotype relevant to supporting various positive health outcomes. The current study seeks to explore how this phenotype is influenced by person and situation factors known to influence eating and consistency in general. In particular, we test the predictive properties of various personality tests and situations on eating consistency measured by the Experience Sampling Method (ESM).

## Methods

### Participants

195 white adult women were initially recruited from the general population of Montreal by local advertisement. Complete data on ESM and personality were available for 139, forming the final sample used for the majority of analysis. The age range was wide (mean years = 44.9, SD = 17.8, ranging from 18 to 75), and, based on body mass index (BMI, kg/m^2^) [60,61], most women were of normal weight (mean BMI = 22.8, SD = 3.2, ranging from 16.6 to 32.7). When only personality questionnaires were analysed in section Factor analysis of personality traits, we used an extended sample, as personality questionnaire data were available for 164 women (mean age in years = 45, SD = 18, mean BMI = 22.8, SD = 3.1). The ethics committee of McGill University approved the protocol. All participants provided written, informed consent before engaging in a series of experiments, and received monetary compensation for their participation. The data were collected as part of a broader study on age differences in affect, emotions, and lifestyle behaviours in women.

### Methods for measuring consistency

Determining an individual’s consistency rate is labour-intensive, as proper measurement involves sampling eating behaviour across multiple days. Early studies objectively measured food intake and reported that some people vary more in day-to-day food intake than others [28,56,62–67]. Other research has analysed repeated dietary recall measures. Here, lack of consistency is often considered as noise ([68–73], reviewed in [74]). Only one study explicitly considered variation, finding that only a third of people remained consistent during the study period [27]. Finally, a few studies asked people to estimate their consistency with single items asking about regularity or similarity of meals [29–32]. While these measures are less accurate, self-assessments of behavioural consistency have nonetheless been shown to relate strongly to objective measures of behavioural consistency [75].

Here, we studied consistency in a dataset [76] gathered with the Experience Sampling Method (ESM). ESM provides, for the same individual, repeated snapshots of a particular eating behaviour in typical everyday contexts in a reliable and valid form (e.g., [77]) and has been used previously in studies of eating behaviours and self-control (e.g., [78,79,80]). ESM provides a reasonable trade-off as a measure of eating consistency, offering more accuracy and ecological validity than single questions [31,32], but being less labour-intensive and intrusive than precise weighing of food intake [28,56] or repeated assessment of food intake with food-frequency questionnaires [27] or daily recalls [70].

### Data Collection with the Experience Sampling Method

The study lasted 19 days, with participants sampling their behaviour every other day. During the 10 ESM observation days, participants were prompted 6 times a day by an electronic beeper to fill out a short paper-and-pencil questionnaire concerning their emotional states, meals eaten, and situational setting in the previous 2 hours. Participants also had to note the time and day of the event. In total, 9365 observations were made and 3950 meal episodes were reported. The number of meal episodes included in the analysis was reduced because of incomplete data (n = 889) and inconsistent timestamps (i.e., first episode taking place 18:00 and second 10:00, n = 281). A large proportion of the remaining 2780 episodes had timestamps completely missing (n = 651). To avoid losing power when time is involved in the analysis, we used the number of the prompt in a day (1–6) as a proxy measure for time in the final analysis, as these indicators were highly related (see section Descriptive statistics of situations). Therefore, 2780 meal episodes were included in the final analysis (20.0 episodes per participant). In the overall dataset, there was little variation for different days of the week—Tuesday had the fewest episodes (13.0%), whereas Saturday had the most episodes (15.6%). Mean percentage per weekday was 14.3%, SD = 0.8%. On the individual level, most participants expectedly had more meal episodes from weekdays than from weekends or holidays. The average ratio (n weekend+n holiday) / n weekday was 0.52 (SD = 0.25). The ratio was more extreme in a few participants with fewer than 10 meal episodes—the ratio was 2.0 for 2 participants, and 0 for 1 participant. Generalized linear mixed models (GLMM) are robust to unbalanced data, and excluding these participants did not change the analysis; we therefore report the analysis that included them. Further details of the ESM procedure, as well as the effects of emotional states have been reported in a previous analysis [76].

#### Developing an ESM measure of eating consistency

**Fig 1:**
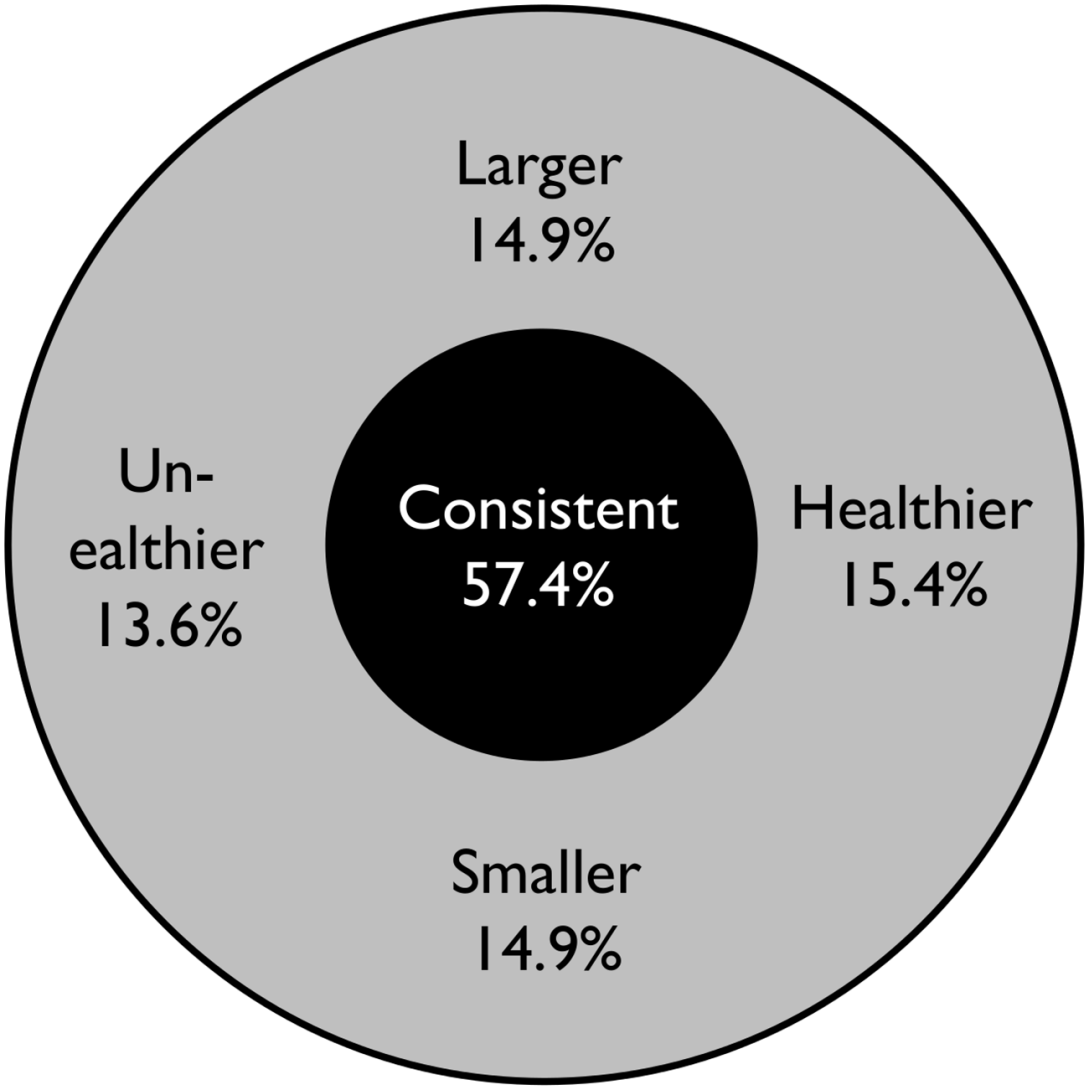
Composite index of consistency. A meal was considered consistent, if it was similar to the regular meal (black circle). If the meal was smaller, larger, healthier, or unhealthier than the regular meal, it was considered inconsistent (grey circle). Percentages denote the rates of various behaviours. The sum of percentages is more than 100% as several inconsistent behaviours could occur at the same time.

To assess eating consistency, we asked participants to rate the perceived nutritional quality and quantity of each meal in comparison with their own corresponding baseline meal for breakfast, lunch, or dinner. The baseline was established during an introductory, face-to-face session. Participants were first asked to describe their own typical food choice for each of the three main meals (breakfast, lunch, and dinner). The experimenter provided information about the nutritional and caloric quality of the food each participant typically consumed on each meal occasion [81]. The experimenter then explained to participants that, throughout the entire study, they should indicate the relative nutritional quality of the meal being reported by comparing that particular meal with the baseline, “typical” meal they described at this introductory session. While people can be systematically mistaken about the actual caloric content, they are still able to judge different meals in terms of their relative caloric content [82]. Therefore, they should be able to tell when meals differ from their regular meal. Detailed characteristics of a typical meal (weekend or weekday, typical situations) were not obtained in the current study as this provided a simpler reference point for participants, and avoided making participants aware of the detailed study objectives. The specific questions asked in ESM were, “In the last 2 hours … If you have eaten a meal, …how does this meal compare to the typical meal you generally take at the same time of the day in terms of composition (i.e., the types of food you had): same as usual, healthier food than usual, or less healthy food than usual.” Similar question was asked about perceived meal size (i.e., same as usual, smaller, or larger than the baseline meal). A meal was considered to be consistent if was similar to baseline in terms of both quality and quantity (coded 1), and inconsistent (coded 0) if not. The composite index is depicted in Fig 1, along with the frequency of various behaviours.

#### ESM measures of situations

Social setting was assessed with two indicators. Participants were asked to indicate the location of the meal they were reporting—responses were coded “0” if they were home, and “1” if they were away from home. When they had eaten a meal within the last 2 hours, they had to indicate if the meal was alone (“0”) or with others (“1”). Information about physical activity and alcohol was gathered with the following questions: “In the last 2 hours… Have you done leisure physical activities not related to work? Have you done physical activities related to work? Have you had an alcoholic drink(s)?” Responses were either “yes” scored as “1” or “no” scored as “0”.

### Questionnaire measures of persons

At enrolment, participants were given a set of questionnaires that they completed at home and returned at the next session. Based on previous evidence that several questionnaires can capture similar underlying mechanisms [83–86], we also expected similar overlap here. The current analysis included various questionnaires known to relate to obesity or other maladaptive eating behaviours [25,41,87–93]: punishment sensitivity (Neuroticism, behavioural inhibition, sensitivity to punishment, emptional eating), reward sensitivity (external eating, reward sensitivity, and Extraversion), and self-control (Conscientiousness, impulsivity, restraint).

The Big Five Inventory (BFI, [94]) is a 44 item questionnaire capturing the broad personality dimensions of the Five-Factor Model. Here three dimensions were included—Neuroticism (α = 0.83), Extraversion (α = 0.82), and Conscientiousness (α = 0.82) that commonly relate to eating behaviours [41,88,95]

Behavioural Inhibition System and Behavioural Activation System Scales (BIS/BAS, [96]) is a questionnaire designed to measure Gray’s behavioural inhibition and behavioural activation systems [97], also known as punishment and reward sensitivity. The BIS scale has 7 items (α = 0.74). The BAS scale divides into three sub-dimensions—reward responsiveness (5 items, α = 0.74), drive (4 items, α = 0.89), and fun-seeking (4 items, α = 0.84), which may provide more detailed insight into the mechanisms of the Behavioural Activation System.

The Sensitivity to Punishment and Sensitivity to Reward Questionnaire (SPSRQ, [98]) is a newer measure of Gray’s traits. Both sensitivity to punishment (α = 0.84) and sensitivity to reward (α = 0.72) subscales have 24 items with yes-no answers. In comparison to BAS scales, the sensitivity to reward scale focuses on specific rewards, whereas BAS scales focus on non-specific rewards [98].

The Barratt Impulsiveness Scale-11 (BIS-11, [99]) is a 30 item widely used impulsivity measure. In the current study, the BIS-11 was inadvertently administered with a yes-no scale instead of the usual 1–4 response scale. Therefore, only the total score was used from the scale (α = 0.77). Nevertheless, the scale maintained its measurement range—the BIS-11 total score replicated known correlations with other scales, such as Conscientiousness (r = -0.52, c.f., [84]), and with impulsive behaviour, such as alcohol consumption (r = 0.27, c.f., [100]). The BIS-11 was included in the study, as impulsivity is an important trait explaining other eating behaviours [41,92].

Weekly alcohol consumption was measured with items from the Canadian Community Health Survey [101]. Participants indicated how many drinks they have had in the previous seven days. The responses were averaged across days (α = 0.72).

The Dutch Eating Behaviour Questionnaire (DEBQ, [102]) is a 33 item questionnaire distinguishing three dimensions: restraint (α = 0.90), external eating (α = 0.84), and emotional eating (α = 0.96). The test was adapted and reproduced by permission of Boom test publishers, Amsterdam, the Netherlands.

The Restraint Scale [103] is another measure of restraint that discriminates between concern for dieting (CD) and weight fluctuation (WF) tendencies. Although the original Restraint Scale was administered, the scoring method of the revised 8 item version (RS-8, [104]) gave better Cronbach alphas (CD: α = 0.67, WF: α = 0.77) in the current study and was therefore substituted.

### Statistical Analysis

To provide an overview of the ESM dataset, we first provide descriptions of consistency, situations, and how they vary in time. We also inspected the relationship between time, prompt number and consistency to demonstrate that prompt number (i.e. 1–6) could be used as a proxy for time.

The many eating-related personality traits that have been identified may in fact reflect a smaller number of shared underlying traits [41,85,86]. To capture the underlying traits, we factor analysed personality trait scores and used factor scores to predict consistency. This approach also reduces the number of potentially multicollinear predictors in a regression model.

Probability of consistent behaviour was predicted by generalized linear mixed models (GLMM with binomial family and logit link). GLMM allows for more flexibility than generalized linear models by allowing a hierarchical data structure. In the current dataset, both consistency and situations have been measured multiple times within person. GLMM allows studying the effect of situations on consistency at each event, while controlling for mean individual differences in consistency across participants. To achieve that, participants are entered into the model as random factors. We ran a step-wise approach, gradually increasing model complexity by adding new parameters or specifications and then determining if the increased model complexity resulted in a substantially better explanation of consistency. The first modelling step was determining the appropriate random intercepts—we tested if the null model would improve if we added random intercepts for persons, accounting for the individual differences in the mean levels of consistency across all episodes. After determining the relevance of random intercepts, the next steps were adding control and personality variables as fixed effects, adding time and situations as fixed effects, adding time and situations as random slopes (i.e., testing if the slope of an effect varies from person to person), and adding two-way interactions. The model complexity-explanatory power trade-off were assessed with Akaike Information Criterion (AIC) and Bayes Information Criterion (BIC)–additional predictors were only retained in the model if the new model resulted in a lower AIC and/or BIC compared to the previous model. To facilitate comparison of the relative effect sizes of continuous personal factors vs binary situations we also present results in a standardized manner as advocated by Gelman [105]–all continuous variables were scaled by dividing them by two standard deviations. All variables were also centered to have a mean of 0 for easier interpretation of interactions.

All analysis was conducted in the R environment 3.2.0 [106]. Factor analysis was conducted with “psych” package [107], general multi-level modelling with “lme4” package command glmer() [108], and subsequent standardizing with “arm” package [109]. For data manipulation and plotting we further depended on packages, such as “plyr”, “sjPlot”, “ggplot2”, “effects”, “streamgraph”, and “gridExtra” [110–115].

## Results

### Individual and time differences in consistency

**Fig 2:**
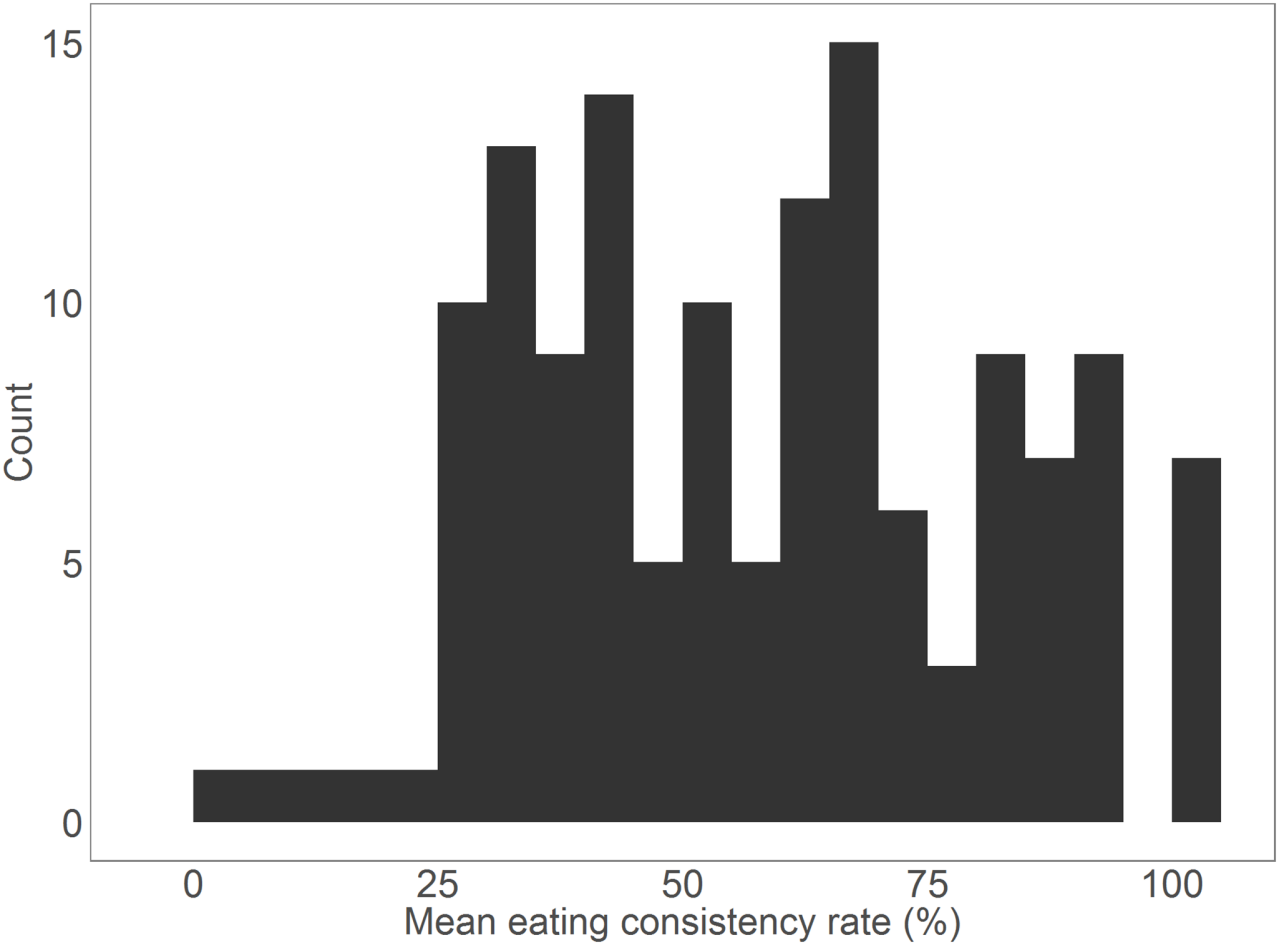
A histogram of the frequencies of different mean eating consistency rates of different individuals, aggregated across all episodes.

First, we tested for individual differences in consistency. To plot these differences (Fig 2), we calculated the mean scores of consistency for each participant, and found considerable variation in consistency (mean = 57.6%, SD = 23.5, ranging from 0–100).

**Fig 3:**
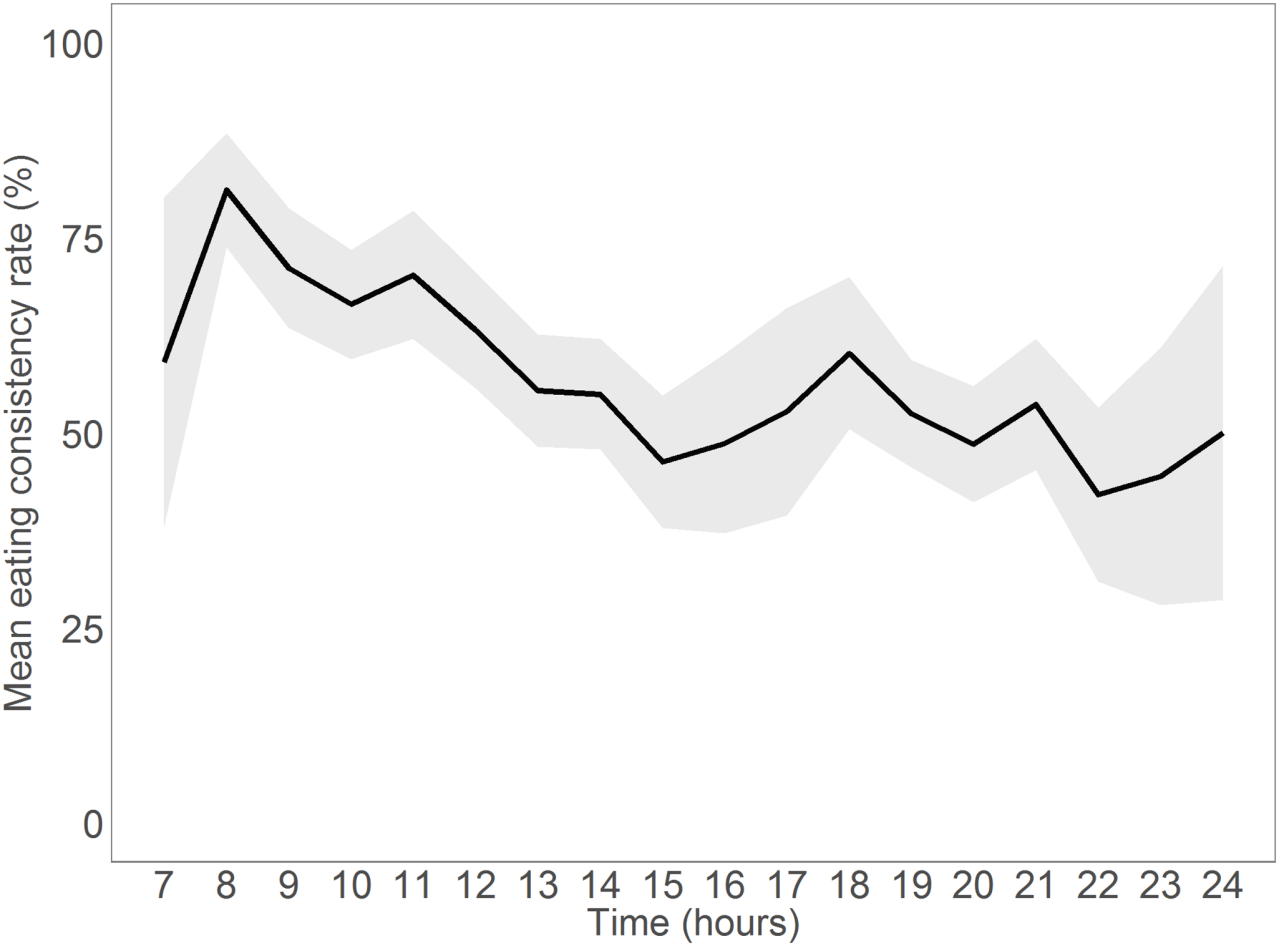
Mean eating consistency rate at each hour from 7AM until midnight, aggregated across all days. Grey area denotes 95% confidence intervals.

**Fig 4:**
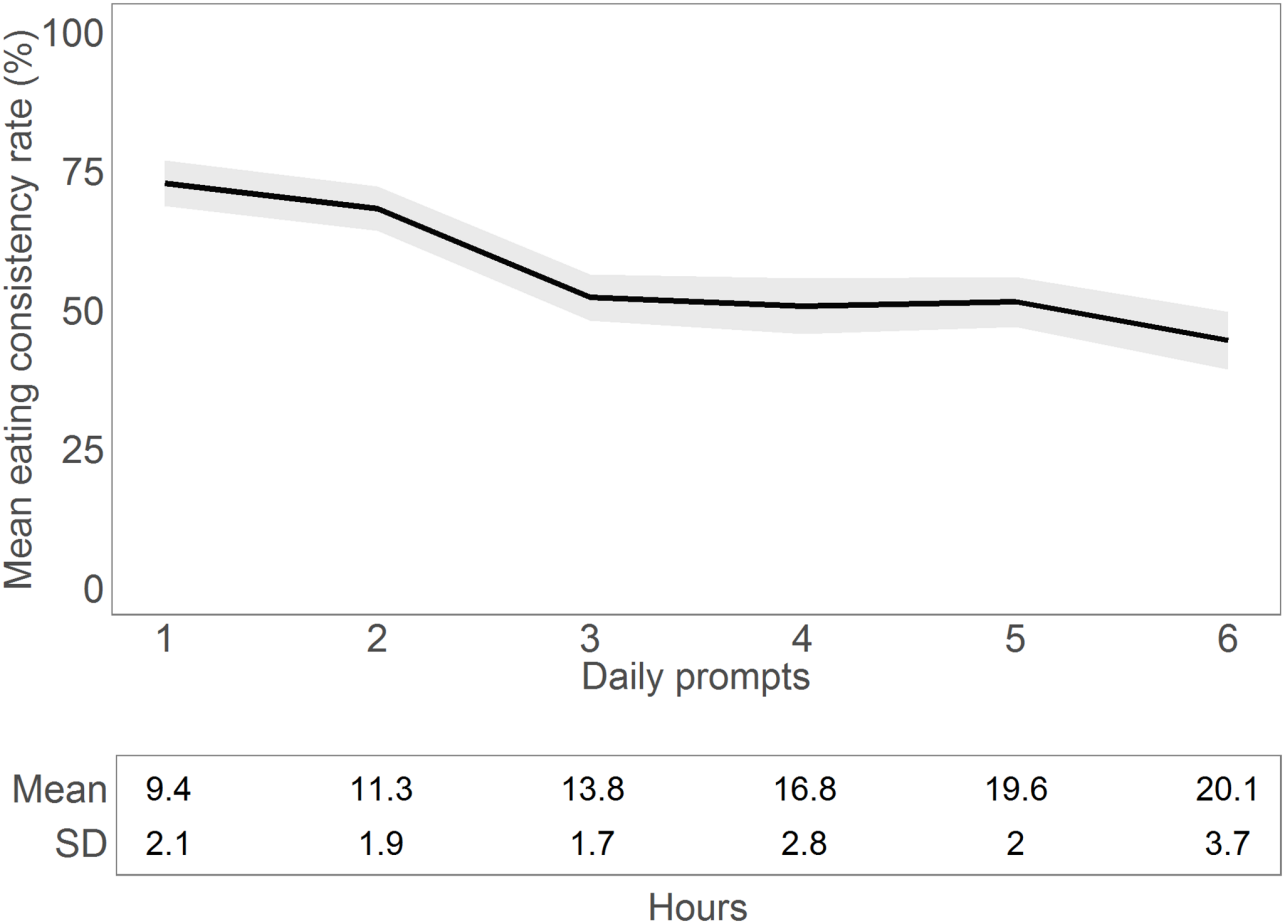
Mean eating consistency rate at prompts 1–6, aggregated across all days. Grey area denotes 95% confidence intervals. Panel below shows the mean time each prompt took place, along with standard deviations (SD). For instance, the mean time when people responded to 1^st^ prompt was 9.4 hours (9:24 AM), with a standard deviation of 2.1 hours (2 hours, 6 minutes).

To inspect the role of time, we aggregated the mean consistency rate for each full hour (7–24) and for each prompt (1–6). We excluded hours 1–6, as these had very few observations (n = 19). As can be seen in Fig 3, consistency starts high in the morning, but there is a considerable decrease thereafter. The decrease is considerably slower in the evening period. As prompt number was highly correlated with time of day (r = 0.85), the prompt plot shows a similar tendency (Fig 4). To account for non-linearity and to reduce data complexity, prompts were recoded into a binary time variable—prompts 1–2 were coded as 0, i.e. morning (mean hour = 10.5, SD = 2.2), and prompts 3–6 were coded as 1, i.e. evening (mean hour = 17.3, SD = 3.6). This morning-evening dichotomy approach is common in other work on the effect of time of day on behaviour (e.g., [116]). After accounting for the effect of morning vs evening on consistency (OR = 0.42, 95% CI [0.36, 0.5]), there was no additional effect of weekday vs holiday or weekend day (OR = 0.88, 95% CI [0.75, 1.04]).

### Descriptive statistics of situations

**Fig 5:**
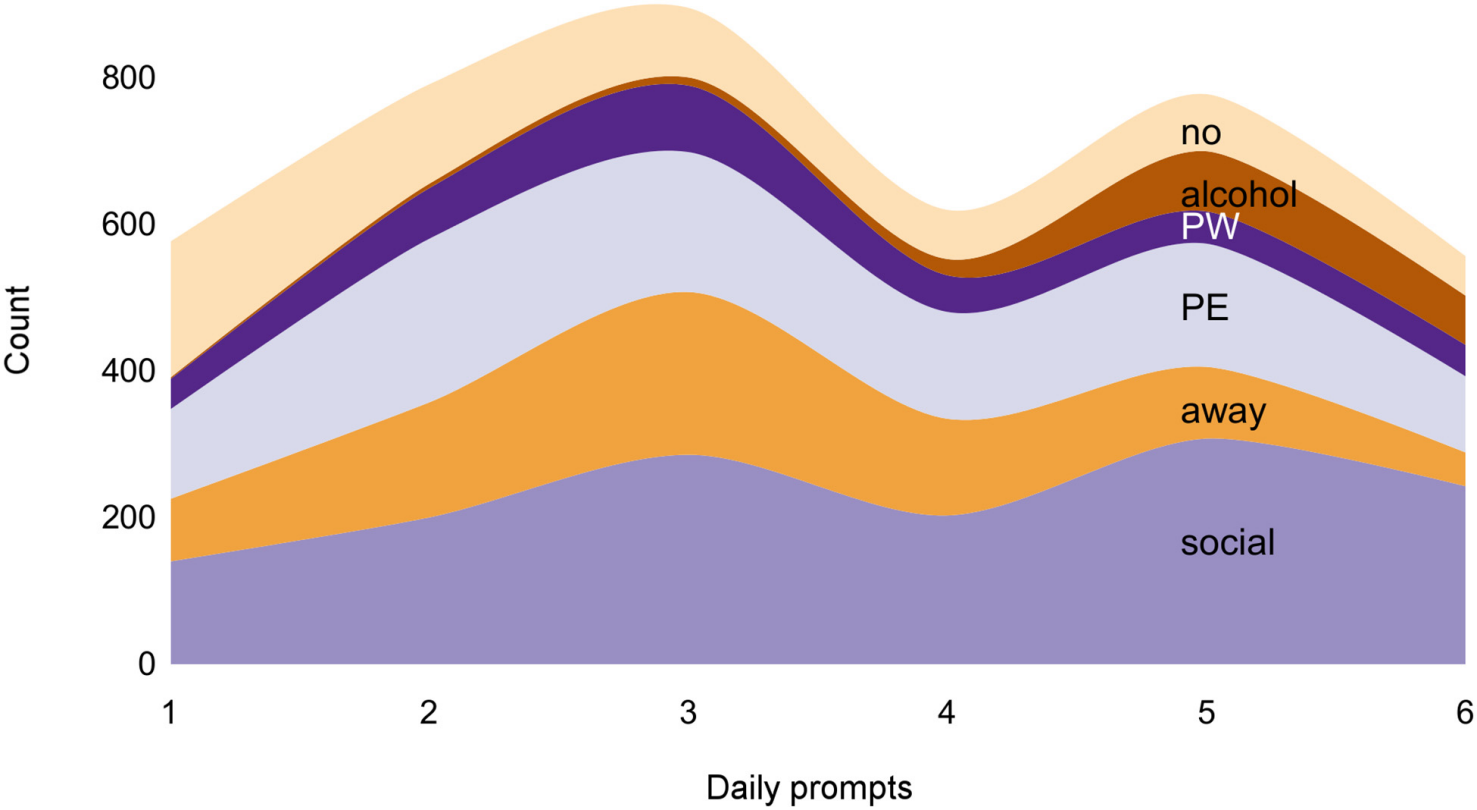
A streamgraph of the frequency of different types of eating situations at prompts 1–6, summed across all days. Alcohol = alcohol was consumed; away = away from home; no = eating-only event; PE = physical exercise; PW = physical work; social = eating with others.

We assessed the prevalence of various types of eating situations—in 49.6% of episodes participants ate with others, in 26.6% episodes they were away from home, in 6.8% of episodes participants had consumed alcohol, in 34.3% episodes they had exercised, and in 12.2% episodes they had undertaken physical work prior to the eating event. In 39.6% of eating episodes, at least two situational factors were present (e.g., away from home and with others). At the same time, 22.2% of eating episodes were eating-only—there was no other situational factor present. Fig 5 shows the count of various types of eating situations at different times of day. We tested if prevalence of these situations was predicted by binary time variable. As suggested by Bandura, drinking alcohol (OR = 14.17, 95% CI [7.43, 31.48]), being away from home (OR = 1.22, 95% CI [1.02, 1.46]), and being in social situations were more likely in the evening (OR = 2.74, 95% CI [2.34, 3.23]), whereas eating-only situations were less likely (OR = 0.91, 95% CI [0.89, 0.94]). Only physical exercise (OR = 0.99, 95% CI [0.84, 1.17]) and physical work (OR = 1.19, 95% CI [0.94, 1.52]) were equally likely throughout the day. Therefore, time is an important covariate when studying the effects of situations.

### Factor analysis of personality traits

Factor analysis was conducted on the sum-scores of individual questionnaires. Parallel analysis suggested the extraction of four factors. However, to segregate the two eating-related factors [41,86], a five-factor solution was preferred, which explained 56% of the variance (Table 1). The first three factors captured self-control (Conscientiousness and impulsivity), punishment sensitivity (Neuroticism, sensitivity to punishment, behavioural inhibition) and reward sensitivity (Extraversion, behavioural activation). The last two factors were eating-specific, the fourth was the combination of emotional and external eating, summarized as Uncontrolled Eating [85], and the fifth a combination of two restraint scales. The weight fluctuation subscale of the Restraint Scale was excluded as it had low communality (18%) and the Sensitivity to Reward scale was excluded as it loaded on many different factors. Factor scores of each factor were extracted for subsequent analysis. The factor loadings and factor scores of self-control were reversed for ease of interpretation.

**Table 1:** Factor loadings and correlation between factor scores.

	Self-Control	Punishment sensitivity	Sensitivity to Reward	Uncontrolled Eating	Restraint
Conscientiousness	**0.80**	-0.20	0.11	0.01	0.00
BIS-11	**-0.65**	-0.13	0.11	0.14	-0.05
Sens to Punishment	-0.08	**0.82**	-0.08	0.00	0.01
BIS	0.00	**0.55**	0.25	0.15	0.04
Neuroticism	-0.25	**0.43**	0.19	0.18	-0.06
BAS Drive	0.08	-0.02	**0.71**	0.03	0.06
BAS Reward Resp	-0.38	-0.22	**0.56**	-0.05	-0.05
BAS Fun Seeking	0.19	0.26	**0.59**	-0.01	0.02
Extraversion	-0.06	-0.33	**0.39**	0.03	0.02
D Emotional Eating	0.04	-0.02	-0.03	**0.86**	0.06
D External Eating	-0.05	0.07	0.03	**0.72**	-0.05
D Restraint	0.09	0.01	0.04	-0.14	**0.80**
Controlled Dieting	-0.11	-0.01	-0.01	0.19	**0.77**
Cumulative variance explained	0.11	0.23	0.34	0.46	0.56
Correlation between factor scores	Self-Control	Punishment sensitivity	Sensitivity to Reward	Uncontrolled Eating	Restraint
Punishment sensitivity	-0.20				
Sens to Reward	-0.11	-0.16			
Uncontrolled Eating	-0.41	0.34	0.17		
Restraint	0.20	0.11	0.08	0.37	

Note: Factor analysis based on minres extraction with oblimin rotation. Bold marks loadings larger than 0.35. Loadings of Self-control have been multiplied by -1 for conceptual clarity. In the factor score correlation matrix, correlations ≥ 0.16 have p < 0.05. BIS = Behavioral Inhibition System scale; BIS-11 = Barratt Impulsiveness Scale-11; BAS = Behavioral Activation System scale; D = Dutch Eating Behavior Questionnaire; Sens = Sensitivity; Resp = responsiveness.

### Predictors of consistency

**Fig 6:**
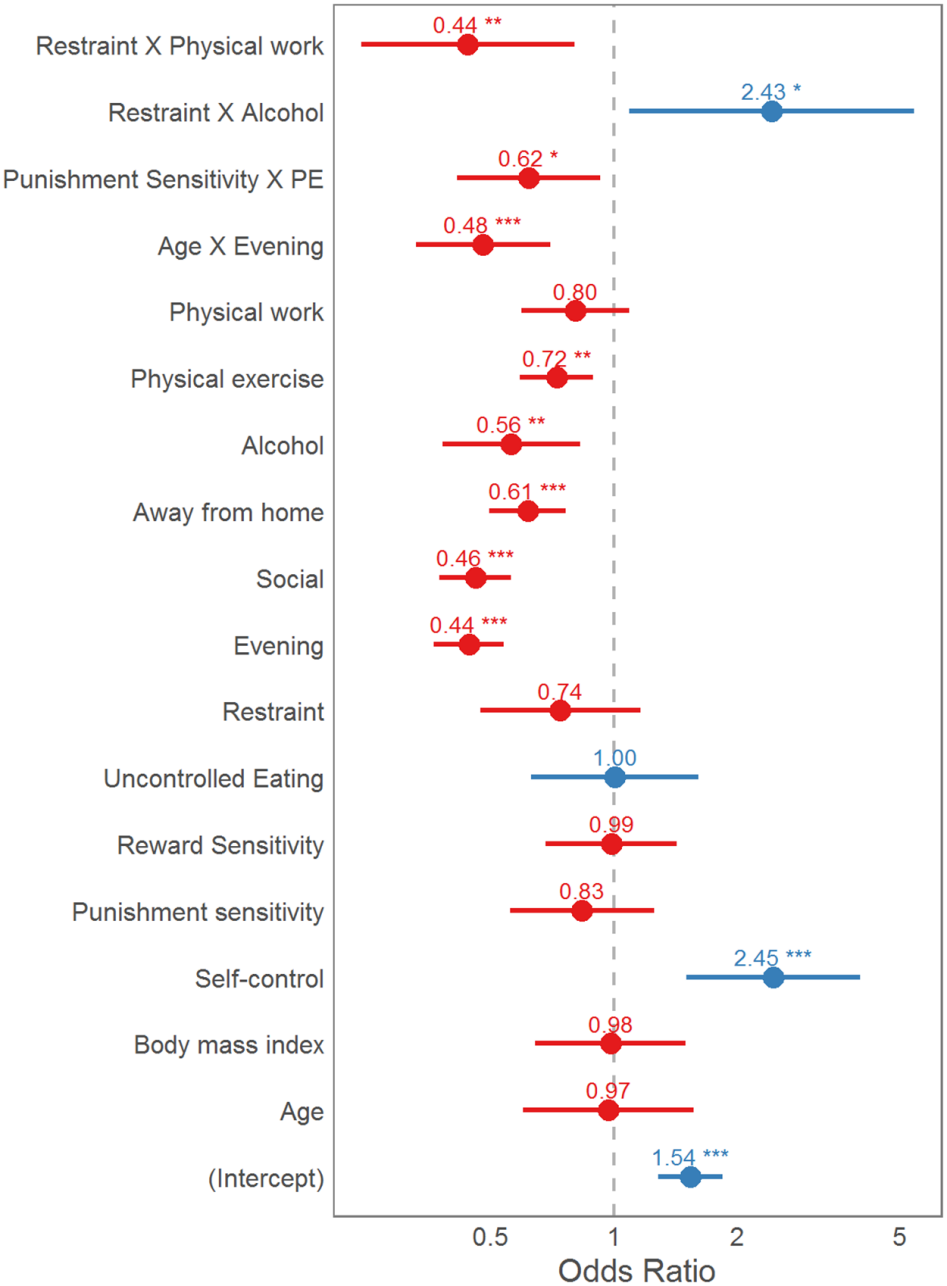
Odds ratios of person and situation predictors of eating consistency. All variables have been standardized as per Gelman [105]–odds ratios denote a change from “no” to “yes” in situations and a 2SD increase in continuous variables. Lines denote 95% confidence intervals. PE = physical exercise. *** = p < 0.001. ** = p <0.01 * = p<0.05.

**Fig 7:**
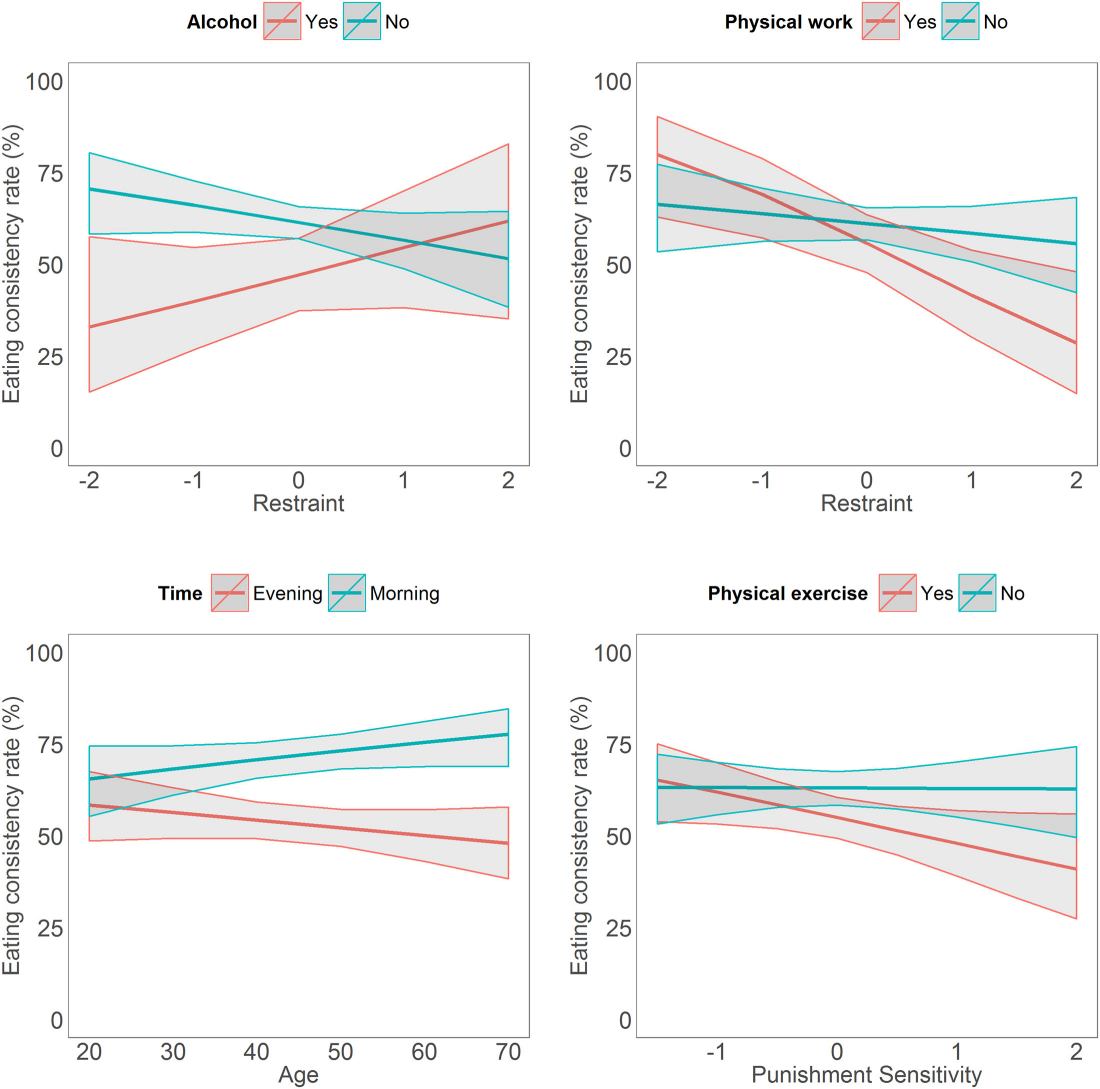
Detailed plots of the interactions in the final model. X axes denotes factor scores of personality traits or age in years, y axis denotes mean eating consistency rate for different situations. Mean value is depicted by bold red/blue line, gray areas denote 95% confidence intervals.

The stepwise procedure for model building is summarised in Table 2. Although some steps increased BIC, they were still included because of theoretical relevance. Only steps decreasing both AIC and BIC were excluded. First, we entered age and BMI as control variables—older people were more consistent (OR = 1.54, 95% CI [1.03, 2.29]). Next, we entered the five factor scores extracted from factor analysis. Self-control positively predicted eating consistency (Fig 6). Adding self-control also caused the age effect to disappear. Thereafter, we turned to situational variables by first adding binary time—morning vs evening and then the social situations. As expected, eating later, eating with others, eating away from home, having consumed alcohol and doing physical exercise negatively predicted consistency (Fig 6). Only physical work episodes had no main effect. Thereafter, we tried modelling time and situations as random slopes for each participant—this model had increased AIC and BIC and therefore this step was excluded (Table 2). A possible explanation could be that situations have similar effects on consistency across people. Finally, we tested all two-way interactions between fixed effects. Only a handful of interactions emerged, and only these interactions were included in the final model (Fig 6). These interactions (Fig 7) highlight that the effects of restraint and punishment sensitivity may only emerge in certain situations. Further, the discrepancy between morning and evening seems to be bigger in older participants.

**Table 2:** Summary of models tested.

Model	Model description	AIC	BIC
M0	Null model	3795.8	3801.7
M1	M0 +random intercepts	3551.7	3563.6
M2	M1 + control variables	3551.3	3575.0
M3	M2 + personality traits	3539.2	3592.5
M4	M3 + time	3430.8	3460.5
M5	M4 + situations	3309.4	3398.3
M6*	M5 + situations as random slopes	3330.0	3579.1
M7	M5 + two-way interactions	3285.1	3397.8

Note. AIC = Akaike Information Criterion. BIC = Bayes Information Criterion. Models with an asterisk were not included in the final model. M0 was estimated with generalized linear models with binomial family, other models were estimated with generalized linear mixed models with binomial family and logit link. The R formula for final model (M7) was: consistency~ control variables + time + traits + situations + four trait-situation interactions + (1|participant ID).

## Discussion

The current study explored how several personality traits and situations can influence consistency in eating, as measured by ESM. As previous studies have noted, there were considerable individual differences in consistency. For the first time, we demonstrated that eating consistency can be influenced by both the personality of the participant and by the situations the participant experienced. Further, several person-situation variables interacted with each other. We hope that understanding the personality and situational factors influencing eating consistency can lead to interventions helping to successfully manage the temptations of today’s eating environment (e.g., [10]).

Self-control has been highlighted as the key variable in various health-related behaviours, including eating [41,90,117,118]. People scoring high in self-control can be expected to have well-ordered lives and to strive to meet their goals in a planned and deliberate manner (e.g., Conscientiousness in [119]). In the current context, people scoring high in self-control are better at keeping their meals consistent. The fact that self-control explained the effect of age is expected, as aspects of self-control are known to improve with age [120]. A possible underlying mechanism of self-control could be better planning capabilities. For instance, recent research in trait self-control has highlighted that the main strategy of successful self-controllers is not fighting off unexpected distractions and temptations. Instead, successful self-controllers tend to design effective habits to avoid being confronted with temptations in the first place [78]. Hence, interventions aimed at improving eating consistency should focus on strategies that help people to avoid being confronted with temptations and distractions.

As expected, people were less consistent in the evening. This is in accordance with the fatigue hypothesis [57], that later in the day people have less self-control capabilities. Importantly, the effect did not interact with trait self-control, suggesting that personality is unable to “protect” from that effect. However, the evening effect was magnified for older women, suggesting that they might be more vulnerable to the fatigue effect. We found no support that people would be less consistent on the weekend.

The undermining role of various social situations has been demonstrated in other contexts and was further cemented here—people eat less consistently when eating with others, away from home, when they have consumed alcohol and when they have exercised. Here, eating differently after exercising could be considered reasonable, as calories have to be restored. Future research capturing objective intake could look for further moderators, as people differ widely in their objective intake response to physical activity [54]. In turn, other situations might lead to unwanted change in food consumption, as alcohol and social situations divert attention away from food, and social situations likely offer food different than usual. While these situations do little harm in isolation, frequent eating in these contexts could have permanent health effects. People looking to improve their eating consistency should be aware of the power of situations. The effects of situations were independent of time, suggesting that both fatigue and the situations independently influence consistency, supporting the models of both Baumeister and Heatherton [57], as well as Bandura [58].

Some situations further interacted with personality traits. An expected interaction was that while unrestrained eaters are less consistent after consuming alcohol, restrained eaters are more consistent. This result mirrors the findings of Polivy and Herman [49,59] who propose alcohol leads to an elevated mood which facilitates less restrained people to restrain even less, and more restrained people to restrain more. An unexpected finding was that higher restraint related to inconsistency after physical work. Usually, restrained persons tend not to compensate after physical exercise [53]. It could be speculated that restrained eaters were rationalizing their lapse in consistency by justifying it by the preceding physical work. However, in this case one would also expect justification with other situations. Participants low in punishment sensitivity were more likely to be inconsistent after physical exercise. A potential explanation could be that less punishment sensitivity (i.e., more anxiety) leads people to be more sensitive to their bodily signals and therefore to be more likely to compensate after intensive physical activity.

Consistency as defined in the current study does not imply having exactly the same food items across different days. Research on sensory specific satiety suggests that people like to vary their diet [121]. In the current study, participants could still be consistent while enjoying different types of fruit, vegetables, or meat. To achieve consistency, the overall meal size or meal healthiness of the meals should be the same. This could be achieved by following the general healthy eating index guidelines (e.g., [12,13]) while mixing individual components.

The current results are a step towards understanding the determinants of eating consistency. Individual differences in consistency are well-known but the psychosocial predictors have not been studied, despite the known health benefits of better consistency. One reason may be the resource-intensive data collection required to repeatedly measure eating behaviours. This limitation is less relevant today, as repeated probing of participants about their behaviour is significantly simplified thanks to the prevalence of smart-phones, with multiple apps available for this purpose [122]. Of course, ESM-based measures of consistency need to be validated against more objective measures of consistency, perhaps by asking participants to take pictures of foods or tracking participants’ food purchases. A low-effort and objective measure of consistency could be as helpful for people making eating decisions, as lane and fatigue trackers are for car drivers.

Knowing consistency-related factors could also be beneficial for research determining the usual diet from dietary recall measures. For instance, Neuhaus et al. [34] concluded that dietary data from older persons are more consistent—based on the current results one could argue that knowing trait self-control would be more informative than age. Another possibility is to collect situational data along with dietary recall data. Such additional data would allow distinguishing between self-directed dietary choices and those that reflect environmental influences. Of course, having various situations present during eating could be part of a participant’s regular life; fully excluding them would provide an incomplete picture of a participant’s dietary patterns. Still, mapping the effects of situations could be beneficial. From an intervention perspective, having situational information could help focus counselling efforts either on a person’s own food preferences or on the effects of situations shifting these preferences.

### Limitations

One potential limitation of the study is that the ESM data were based on participants’ subjective assessment of meal consistency, and the accuracy of these assessments was not estimated. These issues could be addressed in a future study. We note that previous evidence suggests that people are generally able to assess the relative caloric difference in meals, although they might underestimate actual caloric intake [82]. Therefore, the current approach should be a reasonable proxy for detecting deviations from regular meals. Nonetheless, we agree that would be useful to replicate current explorative results using more direct measurements of actual food intake (e.g., [56,64]). Direct measurements would also solve the problem of reference meals—currently we asked participants to focus on their typical meal, which could have been a different meal in different contexts for different participants. Further, objectively capturing food intake would enable more precise analysis of actual deviations—as suggested by Pachucki [27], only certain healthful changes in diet are more beneficial than consistency. To inspect the predictors of these particular healthful changes, more detailed food intake data are needed. Future meal analysis could also incorporate energy density, which is another important aspect influencing caloric intake [123].

Another limitation is the non-standard scoring of BIS-11. However, the correlations of BIS-11 with Conscientiousness and alcohol consumption are similar to already known correlations, suggesting this issue is not of major concern. Participants in this study were mostly non-obese white English-speaking women in good health. The choice of this homogenous sample is justified by its theoretical relevance and the increased power it gives in examining the associations between eating consistency, situations, personality measures and their interactions. However, the lack of obese persons might explain why BMI did not relate to eating consistency—the link between eating consistency and body fat percentage was established in an exclusively obese sample [28]. Prospective work is needed to clarify the longer-term predictive power of the consistency measure used in this study. In addition, the generalizability of the results will have to be tested in samples that vary in gender and culture as well as samples that include children and obese populations.

### Conclusions

Despite these limitations, this study provides several important contributions to eating behaviours research. We demonstrated that people vary in their consistency of eating, as measured by the Experience Sampling Method. Further, we found that consistency can be predicted by both personality traits, situations, and by their interactions. We hope that these findings can inform interventions that would help people maintain their personal diet and be less influenced by external situations. Perhaps one day dieters will have access to personal eating consistency aids as elegant as the ones supporting consistent driving.
